# Supplementary material for: Impact of immediate cryopreservation on the establishment of patient derived xenografts from head and neck cancer patients
Source: J Transl Med. 2021 Apr 28;19:180. doi: 10.1186/s12967-021-02850-1 (PMC8082827; doi:10.1186/s12967-021-02850-1)
Supplement: Supplementary file 1 — Additional file 1: Table S1. Detailed STR profiles of patient and PDX from fresh or cryopreserved growth. [file 12967_2021_2850_MOESM1_ESM.docx]

| Table S1. Detailed STR profiles of patient and PDX from fresh or cryopreserved growth. | | | | | | | | | | | | | | | | | | | | | |
| --- | --- | --- | --- | --- | --- | --- | --- | --- | --- | --- | --- | --- | --- | --- | --- | --- | --- | --- | --- | --- | --- |
|  | **UW-SCC-97** | | | **UW-SCC-130** | | | **UW-SCC-133** | | | **UW-SCC-136** | | | **UW-SCC-137** | | | **UW-SCC-148** | | | **UW-SCC-149** | | |
|  | **Patient** | **P1 (Fresh)** | **P1 (Cryo)** | **Patient** | **P1 (Fresh)** | **P1 (Cryo)** | **Patient** | **P1 (Fresh)** | **P1 (Cryo)** | **Patient** | **P1 (Fresh)** | **P1 (Cryo)** | **Patient** | **P1 (Fresh)** | **P1 (Cryo)** | **Patient** | **P1 (Fresh)** | **P1 (Cryo)** | **Patient** | **P1 (Fresh)** | **P1 (Cryo)** |
| D3S1358 | 16 | 16 | 16 | 14,16 | 16 | 14,16 | 15,16 | 16 | 15,16 | NA | 15 | 15 | NA | NA | NA | 16,18 | 16,18 | 16,18 | 15, 16 | 15, 16 | 15, 16 |
| D21S11 | 30,31 | 30,31 | 30,31 | 28, 32.2, 33.2 | 28, 33.2 | 28, 32.2, 33.2 | 31.2, 32.2 | 31.2, 32.2 | 31.2, 32.2 | NA | 29, 31.2 | 29, 31.2 | NA | NA | NA | 30, 30.2 | 30, 30.2 | 30, 30.2 | 29 | 29 | 29 |
| D18S51 | 13 | 13 | 13 | 14,17 | 14,17 | 14,17 | 15,16 | 15,16 | 15,16 | NA | 16,20 | 16,20 | NA | NA | NA | 15,19 | 15,19 | 15,18,19,22 | 14,16 | 14,16 | 14,16 |
| Penta E | 5,12 | 5,12 | 12 | 7,12 | 7 | 7,12 | 7,12 | 7,12 | 7,12 | NA | 5,7 | 5,7 | NA | NA | NA | 7 | 7 | 5,7 | 13,17 | 13,17 | 13 |
| Penta D | 10,16 | 10,16 | 10,16 | 11,12 | 11,12 | 11,12 | 9,12 | 9,12 | 9,12 | NA | 10,11 | 10,11 | NA | NA | NA | 9,12 | 9,12 | 9,11,12 | 11,12 | 11 | 11 |
| D8S1179 | 13 | 13 | 13 | 9,13 | 13 | 13 | 10,13 | 10,13 | 10,13 | NA | 13,15 | 13,15 | NA | NA | NA | 12 | 12 | 12 | 13 | 13 | 13 |
| FGA | 20,21 | 20,21 | 20,21 | 20,21 | 20 | 20,21 | 21,23 | 21,23 | 21,23 | NA | 22 | 22 | NA | NA | NA | 20,22 | 20,22 | 20,22 | 22,24 | 22,24 | 22,24 |
| D5S818 | 11,12 | 11,12 | 11,12 | 11 | 11 | 11 | 9,12 | 9,12 | 9,12 | NA | 11 | 11 | NA | NA | NA | 12,13 | 12,13 | 12,13 | 12,13 | 12,13 | 12,13 |
| D13S317 | 9 | 9,11 | 11 | 12,14 | 12,14 | 12,14 | 8,11 | 11 | 11 | NA | 8 | 8 | NA | NA | NA | 11 | 11 | 11 | 11,12 | 12 | 12 |
| D7S820 | 9,11 | 9,11 | 9,11 | 9,10 | 9,10 | 9,10 | 12,13 | 12,13 | 12,13 | NA | 11,12 | 11,12 | NA | NA | NA | 10 | 10 | 10 | 10 | 10 | 10 |
| D16S539 | 9,12 | 9,12 | 9,12 | 10,12 | 12 | 10,12 | 12,13 | 12 | 12 | NA | 12,13 | 12,13 | NA | NA | NA | 11,13 | 11,13 | 9,11,13 | 8,11 | 8,11 | 8,11 |
| vWA | 15,16 | 15,16 | 15,16 | 17,18 | 17,18 | 17,18 | 14,18 | 14,18 | 14,18 | NA | 17,20 | 17,20 | NA | NA | NA | 16,17 | 16,17 | 14,16,17 | 17,18 | 17,18 | 17,18 |
| THO1 | 6,7 | 6,7 | 6,7 | 8,9 | 9 | 8,9 | 6,9 | 6,9 | 6,9 | NA | 9.3 | 9.3 | NA | NA | NA | 6,9.3 | 6,9.3 | 6,9.3 | 6,9.3 | 6,9.3 | 6,9.3 |
| Amelogenin | X | X | X | X,Y | X | X,Y | X,Y | X,Y | X,Y | NA | X,Y | X,Y | NA | NA | NA | X,Y | X,Y | X | X | X | X |
| TPOX | 8 | 8 | 8 | 8,11 | 8,11 | 8,11 | 10 | 10 | 10 | NA | 11 | 11 | NA | NA | NA | 8 | 8 | 8 | 8,11 | 8,11 | 8,11 |
| CSF1PO | 12 | 12 | 12 | 10,12 | 10 | 10,12 | 10 | 10 | 10 | NA | 12 | 12 | NA | NA | NA | 10,12 | 10,12 | 10,11,12 | 9,11 | 9,10,11 | 9,11 |
| **Percent Match Algorithm Patient to P1** |  | **100** | **100** |  | **100** | **100** |  | **100** | **100** |  | **100** | | **NA** | **NA** | **NA** |  | **100** | **79** |  | **96** | **96** |
